# The genome sequence of the Summer Chafer, *Amphimallon solstitiale *(Linnaeus, 1758)

**DOI:** 10.12688/wellcomeopenres.21100.1

**Published:** 2024-03-08

**Authors:** Douglas Boyes, Liam M. Crowley, Peter W.H. Holland, University of Oxford and Wytham Woods Genome Acquisition Lab, Darwin Tree of Life Barcoding collective, Wellcome Sanger Institute Tree of Life Management, Samples and Laboratory team, Wellcome Sanger Institute Scientific Operations: Sequencing Operations, Wellcome Sanger Institute Tree of Life Core Informatics team, Tree of Life Core Informatics collective, Darwin Tree of Life Consortium

**Affiliations:** 1 UK Centre for Ecology & Hydrology, Wallingford, England, UK; 2 University of Oxford, Oxford, England, UK

**Keywords:** Amphimallon solstitiale, Summer Chafer, genome sequence, chromosomal, Coleoptera

## Abstract

We present a genome assembly from an individual male *Amphimallon solstitiale* (the Summer Chafer; Arthropoda; Insecta; Coleoptera; Scarabaeidae). The genome sequence is 1,584.1 megabases in span. Most of the assembly is scaffolded into 11 chromosomal pseudomolecules, including the X and Y sex chromosomes. The mitochondrial genome has also been assembled and is 19.29 kilobases in length.

## Species taxonomy

Eukaryota; Opisthokonta; Metazoa; Eumetazoa; Bilateria; Protostomia; Ecdysozoa; Panarthropoda; Arthropoda; Mandibulata; Pancrustacea; Hexapoda; Insecta; Dicondylia; Pterygota; Neoptera; Endopterygota; Coleoptera; Polyphaga; Scarabaeiformia; Scarabaeoidea; Scarabaeidae; Melolonthinae; *Amphimallon*; *Amphimallon solstitiale* Linnaeus, 1758 (NCBI:txid360071).

## Background

The tilt of the Earth’s axis of rotation means that once each year the northern hemisphere experiences the day with the longest period of daylight, which may fall on either 20 or 21 June. Before the summer solstice the hours of daylight are increasing each day; after the summer solstice the hours of daylight are decreasing. Many species of insect have life-history traits attuned to day length changes, a trait known as photoperiodism ( Denlinger *et al.*, 2017). In many cases, there is interplay between photoperiodism and temperature sensitivity, with each contributing to when an insect enters or emerges from diapause or changes behaviour. The cellular and molecular basis of these responses is incompletely understood although progress is being made in studies using Lepidoptera ( Denlinger *et al.*, 2017; Kozak *et al.*, 2019); case studies from other insect orders are needed to further an understanding of this important biological phenomenon.

*Amphimallon solstitiale* (order Coleoptera, family Scarabaeidae) is a species of beetle with a life history that seems exquisitely linked to day length in the northern hemisphere. The larvae feed underground on roots and tubers, spending two or three years as a larva before pupating underground in the final spring ( UK Beetles, no date). In midsummer, notably around the summer solstice, adults emerge to mate. Males gather at the tops of trees and tall shrubs in late evening, or even above chimney stacks on houses, swarming in numbers ranging from a few individuals to over a hundred. Not every tree is chosen with apparent preference for the tallest (PWHH, personal observation). These impressive aggregations are often seen on the evening of the summer solstice itself and may continue until mid-July. After mating, females descend to the ground to lay eggs in the soil. A female sex pheromone has been identified ( Tolasch *et al.*, 2003). The behavioural link to midsummer has given rise to the common name Summer Chafer, European June beetle or the Solstice beetle, and the specific name *solstitiale*.

*A. solstitiale* is found across central and northern Europe including southern regions of England and Wales and southern Scandinavia. Further east, there are abundant records from Russia and scattered records from Georgia, Armenia, Tajikistan, Kyrgyzstan and Uzbekistan ( GBIF Secretariat, 2023).

The underground larva can occasionally reach pest proportions in garden lawns and commercial horticulture ( Royal Horticultural Society, 2023). Proposed control measures include the use of entomopathogenic nematodes; however, microbiota screening suggests that bacteria in the gut of *A. solstitiale* include species antagonistic to symbiotic bacteria of nematodes ( Skowronek *et al.*, 2021).

Here we report a complete genome sequence for the Summer Chafer *Amphimallon solstitiale* determined as part of the Darwin Tree of Life project. The genome sequence of *A. solstitiale* will facilitate research into the molecular control of photoperiodism and contribute to the rapidly growing resources for studying genome evolution in insects.

## Genome sequence report

**Figure 1.**
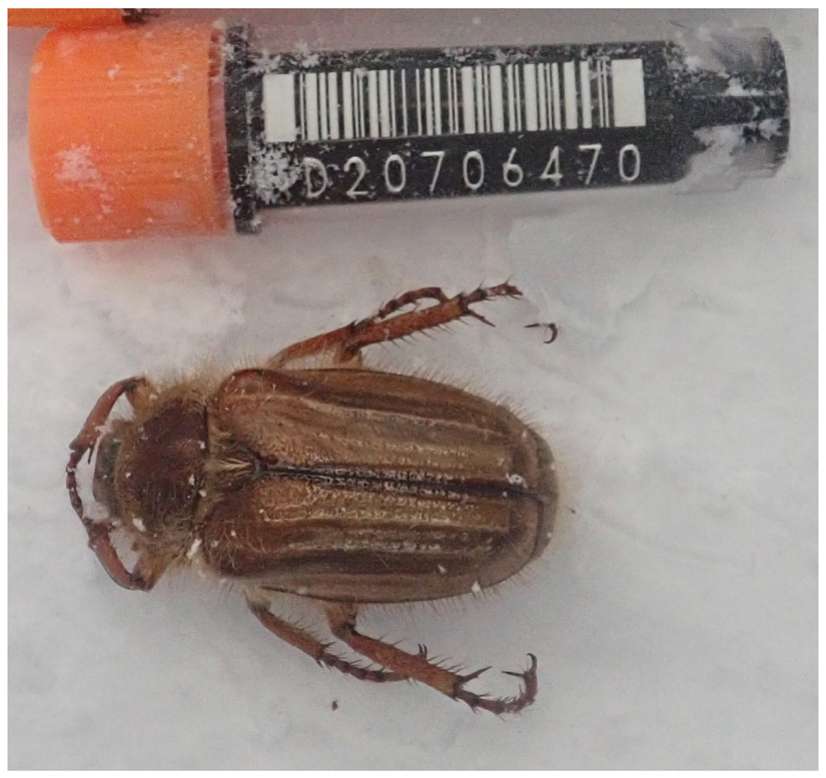
Photograph of the *Amphimallon solstitiale* (icAmpSols1) specimen used for genome sequencing.

The genome was sequenced from one male *Amphimallon solstitiale* ( Figure 1) collected from Wytham Woods, Oxfordshire, UK (51.77, –1.34). A total of 47-fold coverage in Pacific Biosciences single-molecule HiFi long reads was generated. Primary assembly contigs were scaffolded with chromosome conformation Hi-C data. Manual assembly curation corrected 122 missing joins or mis-joins and removed 25 haplotypic duplications, reducing the assembly length by 1.00% and the scaffold number by 6.10%.

**Figure 2.**
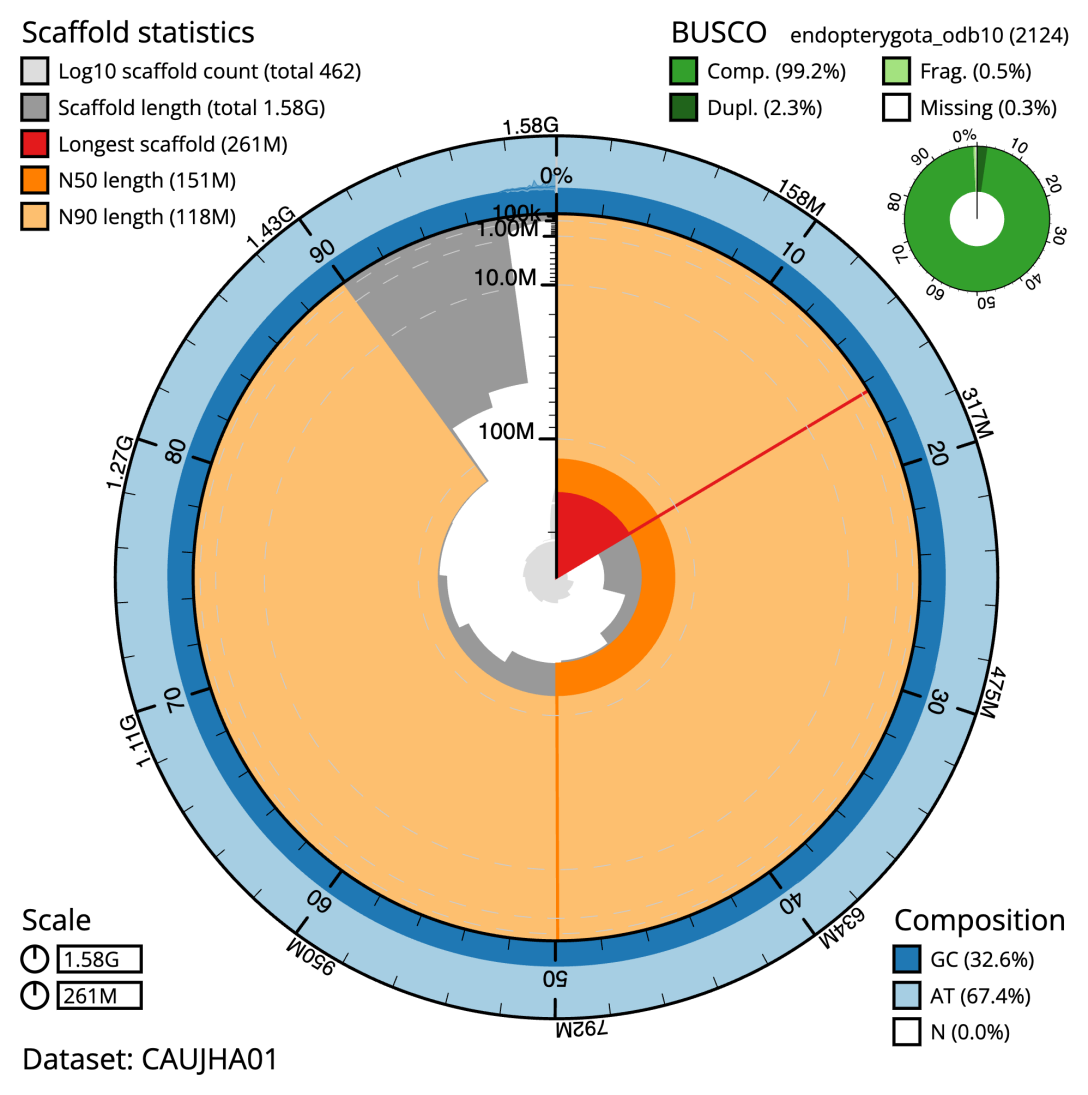
Genome assembly of *Amphimallon solstitiale*, icAmpSols1.1: metrics. The BlobToolKit Snailplot shows N50 metrics and BUSCO gene completeness. The main plot is divided into 1,000 size-ordered bins around the circumference with each bin representing 0.1% of the 1,584,161,395 bp assembly. The distribution of scaffold lengths is shown in dark grey with the plot radius scaled to the longest scaffold present in the assembly (260,577,686 bp, shown in red). Orange and pale-orange arcs show the N50 and N90 scaffold lengths (150,921,111 and 118,082,474 bp), respectively. The pale grey spiral shows the cumulative scaffold count on a log scale with white scale lines showing successive orders of magnitude. The blue and pale-blue area around the outside of the plot shows the distribution of GC, AT and N percentages in the same bins as the inner plot. A summary of complete, fragmented, duplicated and missing BUSCO genes in the endopterygota_odb10 set is shown in the top right. An interactive version of this figure is available at https://blobtoolkit.genomehubs.org/view/CAUJHA01/dataset/CAUJHA01/snail.

**Figure 3.**
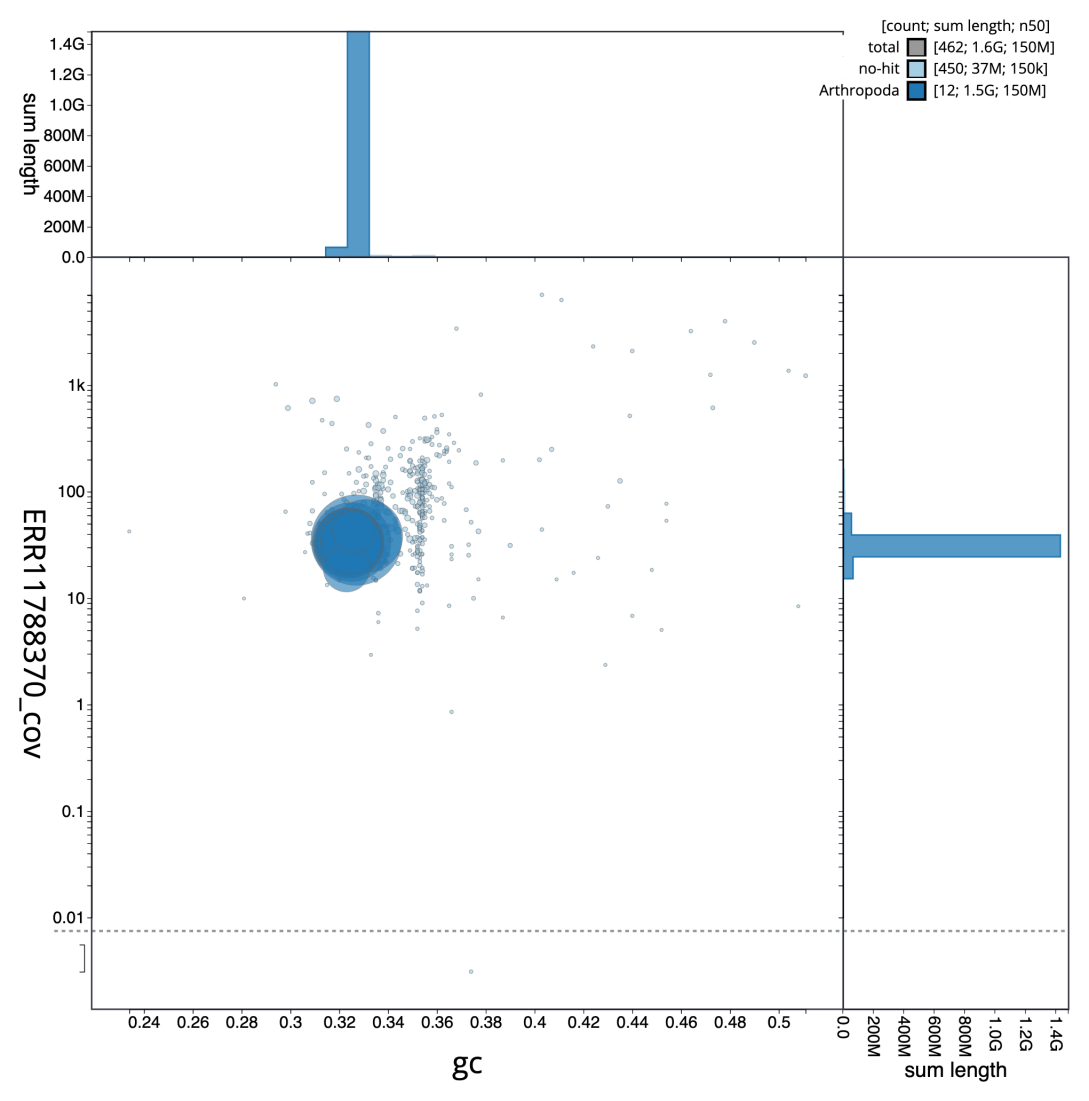
Genome assembly of *Amphimallon solstitiale*, icAmpSols1.1: BlobToolKit GC-coverage plot. Sequences are coloured by phylum. Circles are sized in proportion to sequence length. Histograms show the distribution of sequence length sum along each axis. An interactive version of this figure is available at https://blobtoolkit.genomehubs.org/view/CAUJHA01/dataset/CAUJHA01/blob.

**Figure 4.**
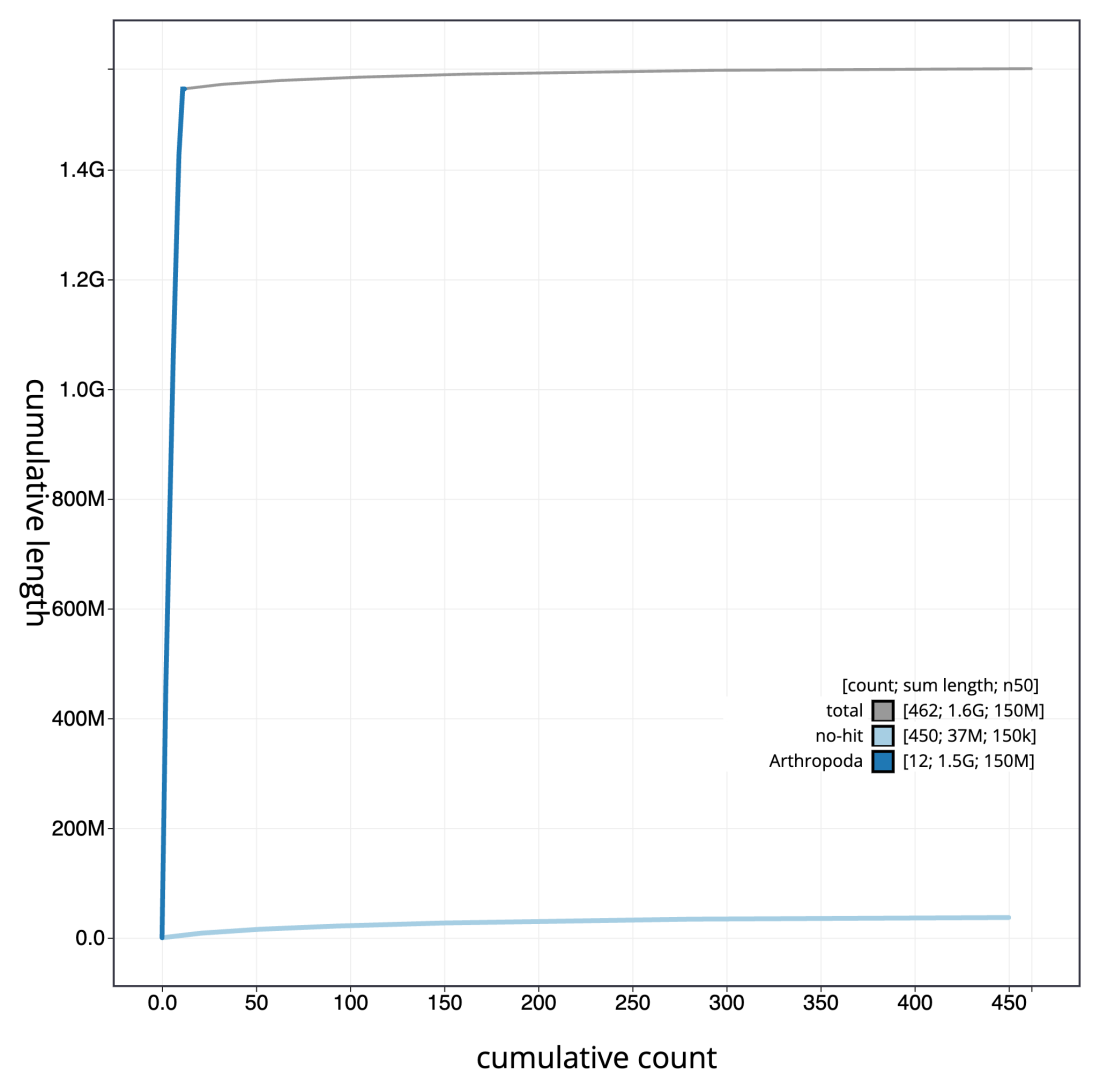
Genome assembly of *Amphimallon solstitiale*, icAmpSols1.1: BlobToolKit cumulative sequence plot. The grey line shows cumulative length for all sequences. Coloured lines show cumulative lengths of sequences assigned to each phylum using the buscogenes taxrule. An interactive version of this figure is available at https://blobtoolkit.genomehubs.org/view/CAUJHA01/dataset/CAUJHA01/cumulative.

**Figure 5.**
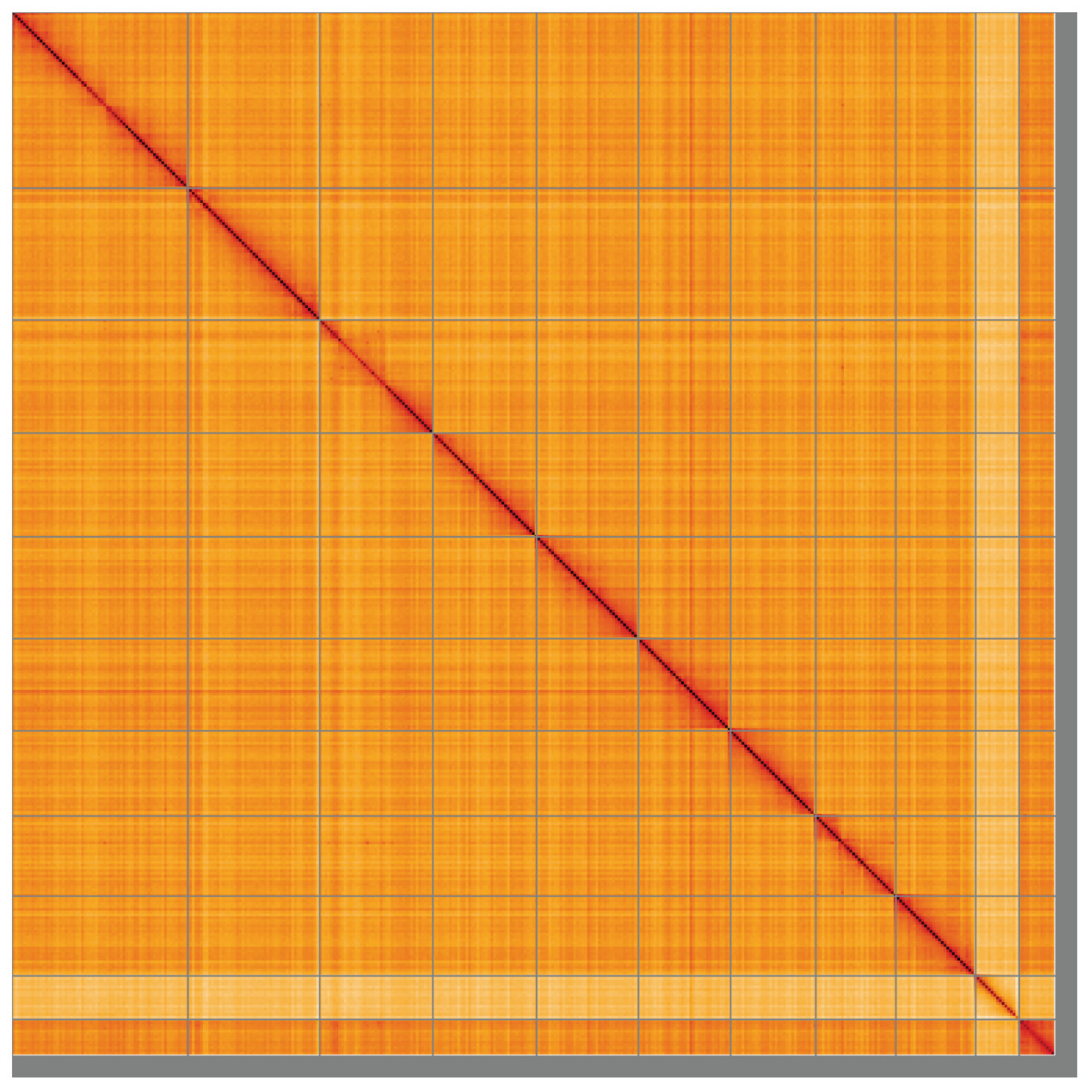
Genome assembly of *Amphimallon solstitiale*, icAmpSols1.1: Hi-C contact map of the icAmpSols1.1 assembly, visualised using HiGlass. Chromosomes are shown in order of size from left to right and top to bottom. An interactive version of this figure may be viewed at https://genome-note-higlass.tol.sanger.ac.uk/l/?d=bmH40SVZR06IKUSWBLb7PA.

The final assembly has a total length of 1,584.1 Mb in 461 sequence scaffolds with a scaffold N50 of 150.9 Mb ( Table 1). The snailplot in Figure 2 provides a summary of the assembly statistics, while the distribution of assembly scaffolds on GC proportion and coverage is shown in Figure 3. The cumulative assembly plot in Figure 4 shows curves for subsets of scaffolds assigned to different phyla. Most (97.69%) of the assembly sequence was assigned to 11 chromosomal-level scaffolds, representing 9 autosomes and the X and Y sex chromosomes. Chromosome-scale scaffolds confirmed by the Hi-C data are named in order of size ( Figure 5; Table 2). Chromosomes X and Y were assigned by read coverage statistics and synteny to the genome assembly of *Melolontha melolontha (*GCA_935421215.2) ( Ashworth *et al.*, 2023). While not fully phased, the assembly deposited is of one haplotype. Contigs corresponding to the second haplotype have also been deposited. The mitochondrial genome was also assembled and can be found as a contig within the multifasta file of the genome submission.

**Table 1.** Genome data for *Amphimallon solstitiale*, icAmpSols1.1.

Project accession data		
Assembly identifier	icAmpSols1.1	
Species	*Amphimallon solstitiale*	
Specimen	icAmpSols1	
NCBI taxonomy ID	360071	
BioProject	PRJEB61997	
BioSample ID	SAMEA10978893	
Isolate information	icAmpSols1: abdomen (DNA and Hi-C sequencing)	
Assembly metrics *		*Benchmark*
Consensus quality (QV)	63.1	*≥ 50*
*k*-mer completeness	100.0%	*≥ 95%*
BUSCO **	C:99.2%[S:96.9%,D:2.3%],F:0.5%,M:0.3%,n:2,124	*C ≥ 95%*
Percentage of assembly mapped to chromosomes	97.69%	*≥ 95%*
Sex chromosomes	XY	*localised homologous pairs*
Organelles	Mitochondrial genome: 19.29 kb	*complete single alleles*
Raw data accessions		
PacificBiosciences SEQUEL II	ERR11458786	
Hi-C Illumina	ERR11439603	
Genome assembly		
Assembly accession	GCA_963170755.1	
*Accession of alternate haplotype*	GCA_963170735.1	
Span (Mb)	1,584.1	
Number of contigs	1,218	
Contig N50 length (Mb)	3.7	
Number of scaffolds	461	
Scaffold N50 length (Mb)	150.9	
Longest scaffold (Mb)	260.58	

* Assembly metric benchmarks are adapted from column VGP-2020 of “Table 1: Proposed standards and metrics for defining genome assembly quality” from Rhie *et al.* (2021). ** BUSCO scores based on the endopterygota_odb10 BUSCO set using version 5.3.2. C = complete [S = single copy, D = duplicated], F = fragmented, M = missing, n = number of orthologues in comparison. A full set of BUSCO scores is available at https://blobtoolkit.genomehubs.org/view/CAUJHA01/dataset/CAUJHA01/busco.

**Table 2.** Chromosomal pseudomolecules in the genome assembly of *Amphimallon solstitiale*, icAmpSols1.

INSDC accession	Chromosome	Length (Mb)	GC%
OY720675.1	1	260.58	32.5
OY720676.1	2	195.32	32.5
OY720677.1	3	167.66	33.0
OY720678.1	4	153.71	32.5
OY720679.1	5	150.92	32.5
OY720680.1	6	136.69	32.5
OY720681.1	7	126.26	32.5
OY720682.1	8	118.77	32.5
OY720683.1	9	118.08	32.5
OY720684.1	X	64.67	32.5
OY720685.1	Y	54.41	32.5
OY720686.1	MT	0.02	29.5

The estimated Quality Value (QV) of the final assembly is 63.1 with *k*-mer completeness of 100.0%, and the assembly has a BUSCO v5.3.2 completeness of 99.2% (single = 96.9%, duplicated = 2.3%), using the endopterygota_odb10 reference set ( *n* = 2,124).

Metadata for specimens, barcode results, spectra estimates, sequencing runs, contaminants and pre-curation assembly statistics are given at https://links.tol.sanger.ac.uk/species/360071.

## Methods

### Sample acquisition and nucleic acid extraction

A male *Amphimallon solstitiale* (specimen ID Ox001707, ToLID icAmpSols1) was collected from Wytham Woods, Oxfordshire (biological vice-county Berkshire), UK (latitude 51.77, longitude –1.34) on 2021-07-17 in a light trap. The specimen was collected by Douglas Boyes (University of Oxford) and identified by Liam Crowley (University of Oxford), and then snap-frozen on dry ice.

The workflow for high molecular weight (HMW) DNA extraction at the Wellcome Sanger Institute (WSI) includes a sequence of core procedures: sample preparation; sample homogenisation, DNA extraction, fragmentation, and clean-up. In sample preparation, the icAmpSols1 sample was weighed and dissected on dry ice ( Jay *et al.*, 2023). Tissue from the abdomen was homogenised using a PowerMasher II tissue disruptor ( Denton *et al.*, 2023a). HMW DNA was extracted using the Automated MagAttract v1 protocol ( Sheerin *et al.*, 2023). DNA was sheared into an average fragment size of 12–20 kb in a Megaruptor 3 system ( Todorovic *et al.*, 2023). Sheared DNA was purified by solid-phase reversible immobilisation ( Strickland *et al.*, 2023): in brief, the method employs a 1.8X ratio of AMPure PB beads to sample to eliminate shorter fragments and concentrate the DNA. The concentration of the sheared and purified DNA was assessed using a Nanodrop spectrophotometer and Qubit Fluorometer and Qubit dsDNA High Sensitivity Assay kit. Fragment size distribution was evaluated by running the sample on the FemtoPulse system.

Protocols developed by the WSI Tree of Life laboratory are publicly available on protocols.io ( Denton *et al.*, 2023b).

### Sequencing

Pacific Biosciences HiFi circular consensus DNA sequencing libraries were constructed according to the manufacturers’ instructions. DNA sequencing was performed by the Scientific Operations core at the WSI on a Pacific Biosciences SEQUEL II instrument. Hi-C data were also generated from remaining abdomen tissue of icAmpSols1 using the Arima2 kit and sequenced on the Illumina NovaSeq 6000 instrument.

### Genome assembly, curation and evaluation

Assembly was carried out with Hifiasm ( Cheng *et al.*, 2021) and haplotypic duplication was identified and removed with purge_dups ( Guan *et al.*, 2020). The assembly was then scaffolded with Hi-C data ( Rao *et al.*, 2014) using YaHS ( Zhou *et al.*, 2023). The assembly was checked for contamination and corrected as described previously ( Howe *et al.*, 2021). Manual curation was performed using HiGlass ( Kerpedjiev *et al.*, 2018) and PretextView ( Harry, 2022). The mitochondrial genome was assembled using MitoHiFi ( Uliano-Silva *et al.*, 2023), which runs MitoFinder ( Allio *et al.*, 2020) or MITOS ( Bernt *et al.*, 2013) and uses these annotations to select the final mitochondrial contig and to ensure the general quality of the sequence.

A Hi-C map of the final assembly was produced using bwa-mem2 ( Vasimuddin *et al.*, 2019) in the Cooler file format ( Abdennur & Mirny, 2020). To assess the assembly metrics, the *k*-mer completeness and QV consensus quality values were calculated in Merqury ( Rhie *et al.*, 2020). This work was done using Nextflow ( Di Tommaso *et al.*, 2017) DSL2 pipelines “sanger-tol/readmapping” ( Surana *et al.*, 2023a) and “sanger-tol/genomenote” ( Surana *et al.*, 2023b). The genome was analysed within the BlobToolKit environment ( Challis *et al.*, 2020) and BUSCO scores ( Manni *et al.*, 2021; Simão *et al.*, 2015) were calculated.

Table 3 contains a list of relevant software tool versions and sources.

**Table 3.** Software tools: versions and sources.

Software tool	Version	Source
BlobToolKit	4.2.1	https://github.com/blobtoolkit/blobtoolkit
BUSCO	5.3.2	https://gitlab.com/ezlab/busco
Hifiasm	0.16.1-r375	https://github.com/chhylp123/hifiasm
HiGlass	1.11.6	https://github.com/higlass/higlass
Merqury	MerquryFK	https://github.com/thegenemyers/MERQURY.FK
MitoHiFi	3	https://github.com/marcelauliano/MitoHiFi
PretextView	0.2	https://github.com/wtsi-hpag/PretextView
purge_dups	1.2.5	https://github.com/dfguan/purge_dups
sanger-tol/genomenote	v1.0	https://github.com/sanger-tol/genomenote
sanger-tol/readmapping	1.1.0	https://github.com/sanger-tol/readmapping/tree/1.1.0
YaHS	1.2a.2	https://github.com/c-zhou/yahs

### Wellcome Sanger Institute – Legal and Governance

The materials that have contributed to this genome note have been supplied by a Darwin Tree of Life Partner. The submission of materials by a Darwin Tree of Life Partner is subject to the **‘Darwin Tree of Life Project Sampling Code of Practice’**, which can be found in full on the Darwin Tree of Life website here. By agreeing with and signing up to the Sampling Code of Practice, the Darwin Tree of Life Partner agrees they will meet the legal and ethical requirements and standards set out within this document in respect of all samples acquired for, and supplied to, the Darwin Tree of Life Project. 

Further, the Wellcome Sanger Institute employs a process whereby due diligence is carried out proportionate to the nature of the materials themselves, and the circumstances under which they have been/are to be collected and provided for use. The purpose of this is to address and mitigate any potential legal and/or ethical implications of receipt and use of the materials as part of the research project, and to ensure that in doing so we align with best practice wherever possible. The overarching areas of consideration are:

• Ethical review of provenance and sourcing of the material

• Legality of collection, transfer and use (national and international) 

Each transfer of samples is further undertaken according to a Research Collaboration Agreement or Material Transfer Agreement entered into by the Darwin Tree of Life Partner, Genome Research Limited (operating as the Wellcome Sanger Institute), and in some circumstances other Darwin Tree of Life collaborators.

## Data Availability

European Nucleotide Archive: *Amphimallon solstitiale* (summer chafer). Accession number PRJEB61997; https://identifiers.org/ena.embl/PRJEB61997 ( Wellcome Sanger Institute, 2023). The genome sequence is released openly for reuse. The *Amphimallon solstitiale* genome sequencing initiative is part of the Darwin Tree of Life (DToL) project. All raw sequence data and the assembly have been deposited in INSDC databases. The genome will be annotated using available RNA-Seq data and presented through the Ensembl pipeline at the European Bioinformatics Institute. Raw data and assembly accession identifiers are reported in Table 1.

## Author information

Members of the University of Oxford and Wytham Woods Genome Acquisition Lab are listed here: https://doi.org/10.5281/zenodo.7125292.

Members of the Darwin Tree of Life Barcoding collective are listed here: https://doi.org/10.5281/zenodo.4893703.

Members of the Wellcome Sanger Institute Tree of Life Management, Samples and Laboratory team are listed here: https://doi.org/10.5281/zenodo.10066175.

Members of Wellcome Sanger Institute Scientific Operations: Sequencing Operations are listed here: https://doi.org/10.5281/zenodo.10043364.

Members of the Wellcome Sanger Institute Tree of Life Core Informatics team are listed here: https://doi.org/10.5281/zenodo.10066637.

Members of the Tree of Life Core Informatics collective are listed here: https://doi.org/10.5281/zenodo.5013541.

Members of the Darwin Tree of Life Consortium are listed here: https://doi.org/10.5281/zenodo.4783558.
