# Supplementary material for: The genome sequence of the Summer Chafer, Amphimallon solstitiale (Linnaeus, 1758)
Source: Wellcome Open Res. 2024 Mar 8;9:138. [Version 1] doi: 10.12688/wellcomeopenres.21100.1 (PMC11112308; doi:10.12688/wellcomeopenres.21100.1)
Supplement: Peer review history

# Reviewer report: Reviewer response for version 1

**DOI:** 10.21956/wellcomeopenres.23340.r79865

**Published:** 2024-05-22

**Authors:** Changlong Shu

**Affiliations:** 1 Chinese Academy of Agricultural Sciences, Beijing, China

The whole experimental design is very reasonable and the data obtained are also very good, which provides sufficient genetic data for the further study of this insect.

 However, the data provided in Figure 2 is too complex. It is recommended to draw statistical charts according to the data category, so that other peers, especially those who are not specialized in bioinformatics research, can quickly and clearly read the information of interest.

Are sufficient details of methods and materials provided to allow replication by others?

Yes

Is the rationale for creating the dataset(s) clearly described?

Yes

Are the datasets clearly presented in a useable and accessible format?

Yes

Are the protocols appropriate and is the work technically sound?

Yes

Reviewer Expertise:

Resource insects and microbiome

I confirm that I have read this submission and believe that I have an appropriate level of expertise to confirm that it is of an acceptable scientific standard, however I have significant reservations, as outlined above.

# Reviewer report: Reviewer response for version 1

**DOI:** 10.21956/wellcomeopenres.23340.r83352

**Published:** 2024-05-22

**Authors:** Phillip Davidson

**Affiliations:** 1 Department of Biology, Indiana University Bloomington, Bloomington, Indiana, USA

In their genome report, Boyes, Crowley, and Holland present a complete and high quality reference assembly for the Summer Chafer *Amphimallon solstitiale. *Overall, the assembly and report are of superb quality and represent a much needed addition for Melolonthinae genomic resources. The rationale as a model for photoperiodism is well described and the methods are sound. Further, the data are accessible online. I have only a handful of minor revision suggestions that I think will clarify the results and significance of this data set, as listed below. 

 Genome Sequence Report:

1. I see the PacBio coverage is stated at 47X coverage, however, please also state the coverage for the HiC data. 
2. Figure 1: Please add a scale bar or if not possible, consider substituting a separate image that includes one. 
3. The genome of Amphimallon solstitiale is remarkably large for a scarab (nearly 1.6Gb), which is very interesting. I think a nice addition to the results would describe what genomic elements compose this enormous expansion (repetitive elements I may guess) and their biological implication. I do not think a formal repetitive element analysis (annotation, classification) is necessary for a data report, but an estimation of what % of the genome is composed of repeats would improve the description of the results. 
4. Figure 2: The BUSCO scores mini-circle plot states the middle shade of green is 99.2% and represents the completion score. However, I think this label should be changed to "single copy (96.9%)" score as the middle shade green does not appear to compose 99.2% of the plot. Further, listing the SC, Duplication, Frag, and Missing is more standard as it distinguishes single copy and duplication composition.  Then, the complete score of 99.2% can be listed separately or perhaps in the legend. 

Methods: 

1. For the clause "snap-frozen on dry ice". Should this instead read "snap-frozen in liquid nitrogen"? Typically dry ice is used for storage and transportation, so I wanted to check. 

Data Availability: 

1. When the gene annotations are complete and if possible, it may be nice to update this report with a link to those files. 

Are sufficient details of methods and materials provided to allow replication by others?

Yes

Is the rationale for creating the dataset(s) clearly described?

Yes

Are the datasets clearly presented in a useable and accessible format?

Yes

Are the protocols appropriate and is the work technically sound?

Yes

Reviewer Expertise:

Genome assembly and annotation, evolutionary-developmental biology

I confirm that I have read this submission and believe that I have an appropriate level of expertise to confirm that it is of an acceptable scientific standard, however I have significant reservations, as outlined above.

# Reviewer report: Reviewer response for version 1

**DOI:** 10.21956/wellcomeopenres.23340.r83369

**Published:** 2024-05-21

**Authors:** Doga Cedden

**Affiliations:** 1 Agricultural Entomology, Department of Crop Sciences, University of Göttingen, Göttingen, Germany; 2 Department of Evolutionary Developmental Genetics, Johann-Friedrich-Blumenbach Institute, Göttingen, Germany

The data note by Boyes et al. presents a high-quality genome assembly from a male *Amphimallon solstitiale*, commonly known as the summer chafer. Although the beetle is not of high economic importance, the high-quality genome will support studies on its interesting biology.

 The quality assessment of the genome sequence is sufficiently detailed. The methods for preparing the specimen, sequencing, and quality control are appropriate and the authors provide enough details for the replication of the study. The genome is made accessible through an appropriate archive service.

 The Background section provides a nice context for the presented genome sequence. One minor suggestion from my side would be to combine the third and fourth paragraphs of the Background section into one paragraph.

Are sufficient details of methods and materials provided to allow replication by others?

Yes

Is the rationale for creating the dataset(s) clearly described?

Yes

Are the datasets clearly presented in a useable and accessible format?

Yes

Are the protocols appropriate and is the work technically sound?

Yes

Reviewer Expertise:

I have expertise in analyzing genomic data from beetle species. Hence, I was able to assess the majority of the manuscript.

I confirm that I have read this submission and believe that I have an appropriate level of expertise to confirm that it is of an acceptable scientific standard.
